# Enhanced radiation-induced immunogenic cell death activates chimeric antigen receptor T cells by targeting CD39 against glioblastoma

**DOI:** 10.1038/s41419-022-05319-1

**Published:** 2022-10-16

**Authors:** Ting Sun, Yanyan Li, Ying Yang, Bin Liu, Yufei Cao, Wei Yang

**Affiliations:** 1 Neurosurgery and Brain and Nerve Research Laboratory, The First Affiliated Hospital of Soochow University, Suzhou, Jiangsu 215006 China; 2 State Key Laboratory of Radiation Medicine and Protection, School of Radiation Medicine and Protection and Collaborative Innovation Center of Radiation Medicine of Jiangsu Higher Education Institutions, Soochow University, Suzhou, Jiangsu China

**Keywords:** Cancer stem cells, Cancer immunotherapy, Radiotherapy

## Abstract

Chimeric antigen receptor (CAR)-T cells directed to solid tumors have been less effective, due in part to the low or lost expression of specific tumor antigens. Herein, we developed a different strategy to enhance CAR-T cell persistence and efficacy by producing a multispecific CAR-T or vaccine based on immunogenic cell death (ICD). We demonstrated that ionizing radiation activates STAT1-IRF1-CD39 axis to upregulate CD39 expression to form an immunosuppressive tumor microenvironment (TME) to enhance radioresistance. CD39 blockade accumulates extracellular ATP, which activates NLRP3 inflammasome in dendritic cells via P2X7 receptor, thereby promoting radiation-induced ICD. Multispecific CAR-T cells in vitro prepared by elevated ICD suppress the growth of xenografts in nude mice. Radiation and CD39 inhibition-induced ICD of glioma stem cells as a vaccine enhance CAR-T expansion in peripheral blood, multifunctionality in the TME, and antitumor effect in a glioma model. The multispecificity of CAR-T cells, targeting CAR and tumor antigens, vastly enhances the function of conventional CAR-T cells, stimulates a native immune response, and overcomes obstacles of specific antigen loss or low expression of target cells in antitumor therapy.

## Introduction

Glioblastoma multiforme (GBM) is resistant to conventional therapies and evades the innate and adaptive antitumor immune response [1]. Glioma stem cells (GSCs) are a small and rare subpopulation with self-renewing capacity, tumor-propagating potential, and embryonic or tissue stem cell gene expression, and are responsible for cancer maintenance and recurrence [2]. Cancer stem cells are protected from immunologic pressure by constitutive characteristics and activity of stemness, and the coordination of cancer stem cells and different types of immune cells contributes to a more suppressive immune microenvironment to promote malignant growth [3]. Chimeric antigen receptor (CAR)-T cell therapy targeting epidermal growth factor receptor variant III (EGFRvIII) is one of currently progressing clinical trials for GBM [4–6]. However, the progress and popularization of CAR-T therapy for GBM were limited.

CAR-T cells directed to solid tumors have been less effective, due to low or lost expression of specific tumor antigens on the surface of target cells, highly immunosuppressive tumor microenvironment (TME), and the presence of inhibitory factors, cytokines, and immune cells [7]. Cancer vaccines, which are designed to amplify tumor-specific T-cell responses, are a key tool of effective cancer immunotherapy [8, 9]. Effective cancer vaccines should provide tumor antigens along with activation signals to stimulate endogenous tumor-specific T-cell and trigger tumor-specific immune responses [10]. Previous studies demonstrated the potential of cancer vaccination to enhance the persistence of CAR-T cells by preparing CAR-T cells from cytomegalovirus-specific endogenous lymphocytes [11], introducing a CAR together targeting native receptors [12], vaccinating recipients against one antigen of dual-specific CAR-T cells [13], directly boosting vaccine donor cells [14] and using nanoparticulate RNA vaccine for body-wide delivery of the CAR antigen into lymphoid compartments [15]. Therefore, combinatorial approaches of CAR-T therapy and vaccine might be promising to cure a high fraction of patients.

Immunogenic cell death (ICD) is a kind of cell death elicited by a series of anticancer agents including various chemotherapeutics and radiotherapy. The cells under ICD status have fully showed potential as a preventive and therapeutic vaccine for successful antitumor therapy [10, 16, 17]. CD39, also known as ecto-nucleoside triphosphate diphosphohydrolase-1 (ENTPD1), is a surface-expressed enzyme for hydrolyzes Extracellular ATP (eATP), an “eat me” signal of ICD, and carries out critical biological functions in generating immunosuppressive TME [18, 19]. Preclinical studies indicated that blocking CD39 activity enhances the immune cell effect to several cancer cell lines and mouse models [20, 21], which may be an effective strategy to prevent the hydrolysis of immunogenic ATP and regulate immunosuppressive TME.

Native antigen specificities of polyclonally activated CAR-T cells show less effect in clinic. CAR-T-cell expansion in vivo is dependent on CAR ligation and lymphodepletion to provide homeostatic cytokines [12]. In this research, we found that ionizing radiation (IR) upregulated CD39 expression by STAT1-IRF1 pathway. The increased CD39 reduced IR-induced extracellular ATP accumulation, therefore, CD39 inhibition promoted IR-induced ICD. Aiming at the emergence of negative and low expressions of tumor antigen variants in a solid tumor, especially spontaneous EGFRvIII loss as well as antigen escape on EGFRvIII-specific CAR-T cells therapy in human gliomas [6], we evaluated a different strategy to enhance CAR-T-cell persistence and efficacy. To verify the hypothesis that ICD would provide in vivo endogenous stimulation of CAR-T cells via the native T-cell receptor (TCR), we administrated ICD-generating cells to mice bearing glioma or stimulated ICD generation in vivo as a vaccine. The immunogenicity of this ICD vaccine with CD39 inhibition was raised by increasing eATP concentration post-IR. The proliferation and antitumor effect of CAR-T cells were elevated after vaccine inoculation due to boosted ability of CAR-T to recognize tumor stem cells, illustrating the therapeutic potential of ICD activating CAR-T cells for GBM.

## Results

### Radiation of high dosage induces the generation of ICD

**Fig. 1:**
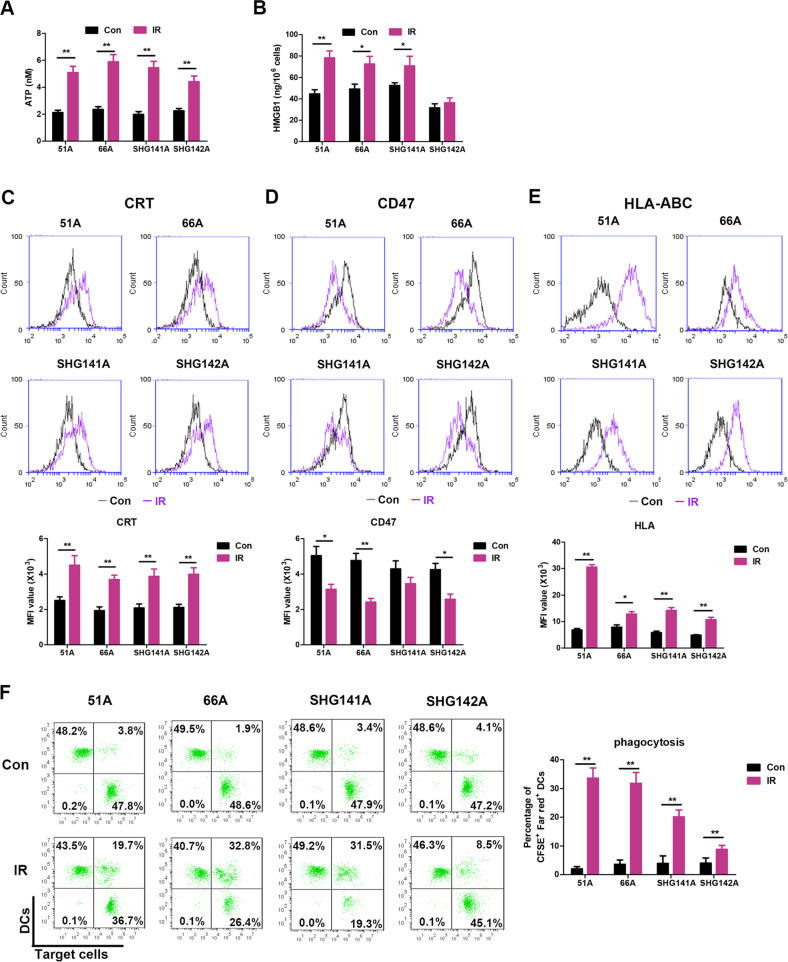
High-dose IR triggers ICD in GSCs. **A**–**E** GSCs were irradiated with dose of 10 Gy, then ICD hallmarks were analyzed 24 h post-IR. **A** Supernatants were collected, and the amount of eATP release was measured by luciferase-based test. **B** Released HMGB1 level was assessed by an ELISA assay in cultured supernatants. The surface exposure of CRT expression (**C**), CD47 expression (**D**), and HLA-ABC expression (**E**) on cell surface were determined by flow cytometry, and mean fluorescence index (MFI) was quantified. **F** Untreated or irradiated GSCs were co-cultured with DCs for 24 hours at a 1:1 ratio, and the percentage of DCs phagocytosis was assessed by flow cytometry. Data represent the mean ± SEM of triplicates and are representative of at least three independent experiments or are plotted as individual points. *P < 0.05, **P < 0.01 vs control.

The emission of ICD hallmark molecules from non-GSCs is radiation dose-dependent and significantly increased following 10 Gy IR [22]. The potential of IR to induce the ICD features was showed in Fig. 1A-C. IR induced increases of eATP, CRT on cell surface and HMGB1 secretion in all detected cells except HMGB1 on SHG142A cells. CD47 molecule, a signal of “do not eat me”, on the cell surface was decreased in 51 A, 66 A cells, and SHG142A after IR (Fig. 1D). In addition, the increase of HLA-I for immune cell recognition was showed following IR (Fig. 1E). Altogether, these results demonstrated that IR triggered cell death characterized by the induction of multiple ICD hallmarks.

We next examined DCs activity to determine whether the immune stimulatory signals induced by IR could enhance DCs phagocytosis. Strikingly, monocyte-derived DCs phagocytized significantly more IR-treated GSCs compared to control (Fig. 1F). Flow cytometric analysis showed that DCs swallowing IR-treated GSCs increased the expression levels of co-stimulatory markers CD83 and CD86, and of HLA, respectively, thus indicating an increased potential to stimulate T-cell activation (Supplementary Fig. 1A). Moreover, expression of the pro-Th1 cytokine IL-12 was stimulated in DCs (Supplementary Fig. 1B), whereas levels of the pro-Treg cytokine IL-10 remained unchanged (Supplementary Fig. 1C), supporting that high dose IR stimulated the phagocytic potential and a pro-inflammatory phenotype appearance of DCs. Altogether, these results indicated that the antigen presentation was activated in GSCs following IR.

### IR induced CD39 upregulation in a STAT1-dependent manner

**Fig. 2:**
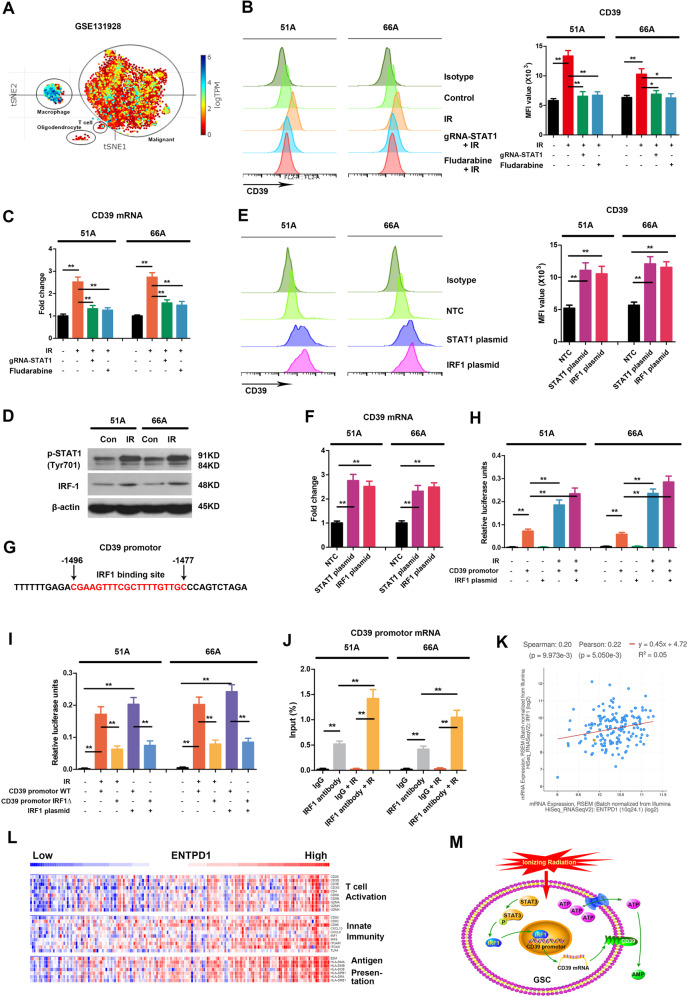
STAT1 mediates radiation-induced CD39 expression via STAT1-IRF1 pathway. **A** t-SNE generated from published data of single-cell RNA sequencing showed relative expression levels of ENTPD1, encoding CD39, in different clusters of human GBM. 51 A and 66 A cells were transfected with plasmid with gRNA targeting STAT1 for 48 h or pretreated with fludarabine, a STAT1 pathway inhibitor, 30 μM for 6 h, then were irradiated with 10 Gy. The level of CD39 protein on cell surface was detected using flow cytometry 24 hours following IR (**B**), and mRNA expression was measured using RT-qPCR 12 hours after IR (**C**). **D** Cells were irradiated with 10 Gy, and the protein levels of phosphorylated STAT1 and IRF1 were tested using western blot 12 hours after IR. Cells were transfected with NTC, pc-DNA-STAT1 or pc-DNA-IRF1 expression plasmids, then CD39 protein (**E**) and mRNA (**F**) expressions were determined. **G** The nucleotide sequence of putative IRF1 binding site in CD39 promotor was shown in red. **H** After 48 h transfection with luciferase reporter pGL3 plasmid carrying CD39 promotor, the luciferase activities in cell lysate were measured by dual-luciferase reporter system when cells were irradiated with 10 Gy or co-transfected with IRF1 expression plasmid. The firefly luminescence signal was normalized based on the Renilla luminescence signal. **I** After transfection with pGL3-CD39 promotor WT or pGL3-CD39 promotor IRF1∆ plasmid, the luciferase activities were measured following IR or IRF1 plasmid transfection. **J** ChIP assay was performed using anti-IRF1 antibody, the results of qPCR products were shown using primers flanking the IRF1 binding site. **K** Correlation of CD39 and IRF1 expressions from TCGA database was analyzed in GBM patients. **L** The expressions of signature immune gene sets based on ENTPD1 expression levels from The Cancer Genome Atlas (TCGA) database were shown using heatmap according to z-score normalized log-cpm values. *n* = 170. **M** IR activates STAT1-IRF1-CD39 axis to form an immunosuppressive TME. *P < 0.05, **P < 0.01.

Hydrolysis of eATP by CD39 and CD73 generates immunosuppressive adenosine to prevent excessive inflammation and tissue damage [18]. Analysis of published single-cell RNA sequencing datasets of human GBM [23] showed high enrichment of ENTPD1 expression in macrophages and moderate enrichment in malignant tumor cells (Fig. 2A). Next, we measured CD39 expression in GSCs after radiation. The result showed increased CD39 protein level on cell surface using flow cytometry (Fig. 2B) and mRNA expression using RT-qPCR analysis (Fig. 2C, Supplementary Fig. 2A, B) following IR.

Radiation activates type I IFN pathway and phosphorylates signal transducers and activators of transcription 1 (STAT1) in host-defense by IFN production, which is the major contributor response to tissue damage. Intrinsic tumor cell radioresistance is one of the main obstructions of effective radiotherapy. Meanwhile, recent report showed that activation of STAT1 is associated with cancer stemness increase [24]. Consistent with previous studies [25], Fig. 2D (Supplementary material 2) showed increase of STAT1 phosphorylation after IR. To investigate whether IR-induced CD39 elevation is regulated by STAT1 pathway in radioresistant GSCs, we knockout STAT1 by deleting part of the gene in exon 3 and 4 including 967 bp using CRISPR/Cas9 gene editing (Supplementary Fig. 2C, Supplementary material 2). Both STAT1 specific inhibitor and STAT1 knockout blocked IR-induced CD39 protein elevation in 51A, 66A, and SHG141A cells (Fig. 2B and Supplementary Fig. 2D), and consistent results were showed in CD39 transcription in 51A and 66A cells (Fig. 2C and Supplementary Fig. 2E). These results suggest that IR regulates CD39 transcription via STAT1 pathway.

### IRF1 binds to the promoter of CD39

The activation of STAT1 pathway leads to the expressions of interferon regulatory factor 1 (IRF1), a downstream transcription factor [26]. To address whether STAT1 and IRF1 could elevate CD39 transcription, GSCs were transfected with or without the STAT1 or IRF1 expressing plasmid. Figure 2E, F showed increased activation of endogenous CD39 after each transfection. Analysis of the CD39 promoter sequence showed putative binding sites for IRF1 (Fig. 2G), but not for STAT1. Thus, we cloned CD39 promotor genomic fragments into firefly luciferase reporter plasmid. IRF1 plasmid transfection showed higher luciferase activity than the non-targeting control (Fig. 2H). Furthermore, we performed site-directed mutagenesis to delete the IRF1 putative binding sites in a CD39 promoter luciferase reporter plasmid. Deletion of the IRF1 site in CD39 promotor reporter plasmid dramatically reduced the luciferase activity (Fig. 2I). To verify the binding of IRF1 to CD39 promotor, ChIP assay was performed using IRF1 antibody. The result in Fig. 2J showed an increased qPCR product when GSCs were transfected IRF1 plasmid, confirming IRF1 binding to CD39 promoter, indicating IRF1-dependent transcriptional activation on CD39. In addition, RNA sequencing analysis from The Cancer Genome Atlas (TCGA) database also shows a positive correlation of IRF1 and CD39 mRNA expressions in GBM (Fig. 2K). Moreover, a strong correlation was showed in ENTPD1^high^ GBM patients for the upregulation of genes involved in T-cell activation, innate immunity, and antigen presentation (Fig. 2L). These data suggested that IR activates STAT1-IRF1-CD39 axis to form an immunosuppressive TME (Fig. 2M).

### Increased eATP-activated NLRP3 in inflammasome of DCs by P2X7 receptor

**Fig. 3:**
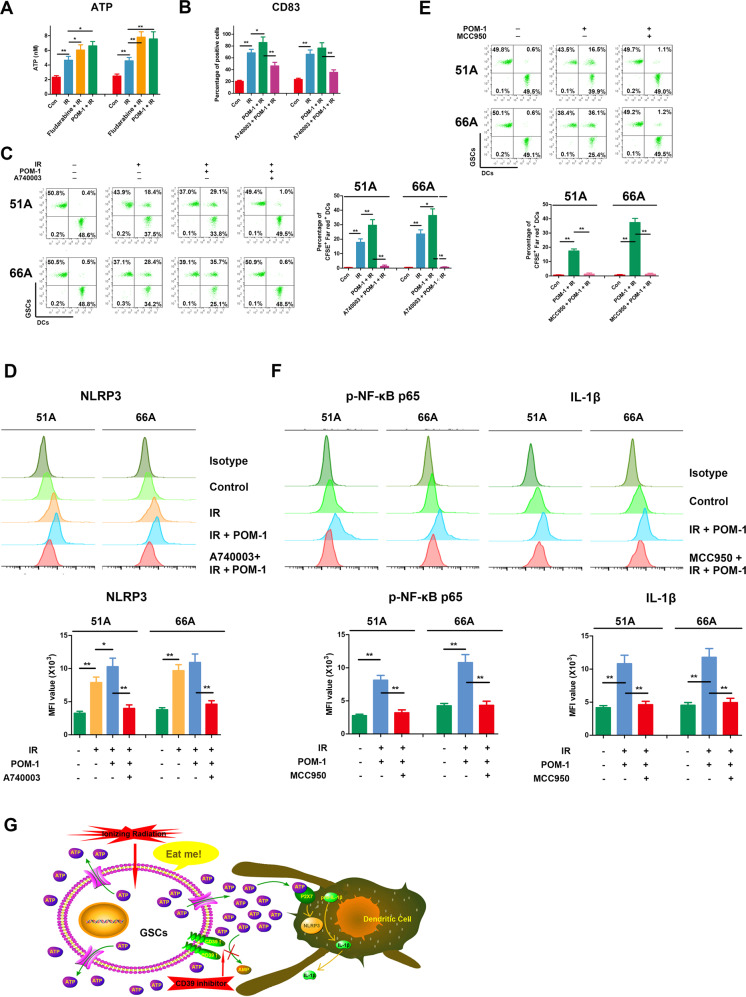
Combination of IR and CD39 inhibition on GSCs activated DCs by NF-κB p65-NLRP3-IL-1β axis. **A** Cells were pretreated with or without 30 μM fludarabine, a STAT1 pathway inhibitor, for 6 h or 100 μM POM-1, a CD39 inhibitor, for 3 h, then irradiated with 10 Gy and cultured for 48 h. Supernatants were collected for ATP measurement. **B** GSCs were labeled by CFSE, and DCs were labeled by Far Red. GSCs were irradiated with 10 Gy in existence or absence with POM-1 and cultured for 48 h, then co-cultured with DCs for 24 h at a ratio of 1:1 with or without 20 μM A-740003, an ATP receptor inhibitor, pretreatment for 1 h. CD83, a mature DCs marker, on the cell surface was stained and detected using flow cytometry. **C** Double-positive cells of CFSE and Far Red were DCs that engulfed GSCs. **D** Supernatants from irradiated GSCs were added into DCs for 24 h, then DCs were collected and NLRP3 level was detected by flow cytometry. **E** GSCs were pretreated with radiation and POM-1 for 48 h, then co-cultured for 24 h with DCs pretreated with or without 10 μM MCC950, an NLRP3 inhibitor, for 30 min. GSCs were labeled by CFSE, and DCs were labeled by Far red. DCs engulfment was measured using flow cytometry. **F** DCs were treated with supernatants from pretreated GSCs, and the expressions of phospho-NF-κB p65 and IL-1β were detected using flow cytometry. **G** Accumulated eATP by CD39 blockade activates NLRP3 inflammasome by P2X7 receptor in DCs. *P < 0.05, **P < 0.01.

Fig. 3A showed that STAT1 inhibition increased ATP release induced by IR, and CD39 inhibitor POM-1 also promoted the effect of IR in GSCs. The ICD-associated release of ATP, a strong chemoattractant, promotes the recruitment and differentiation of immature antigen-presenting cells, an effect depending on purinergic receptor P2X7 [27]. In ICD process, apoptotic bodies and corpses of tumor cells are swallowed by DCs, and human DCs express the P2X7 receptor to a very high level [27], therefore DCs from PBMCs was used to evaluate the effect of immune cells activation. We demonstrated POM-1 treatment on GSCs significantly increased DCs maturation (Fig. 3B) and engulfment on irradiated GSCs (Fig. 3C). A-740003, a selective antagonist of the P2X7 receptor, blocked the effect of IR and POM-1 on DCs engulfment and maturation (Fig. 3B, C), suggesting that increased eATP by CD39 inhibition was needed for DCs activation by surface P2X7 receptor. Extracellular ATP promotes the activation of the NLR family in antigen-presenting cells, stimulating the processing and release of interleukin (IL)-1β and IL-18 [28]. Therefore, we examined the role of NLRP3 inflammasome on DCs activation in increased eATP. Combination of IR and POM-1 treatment on GSCs increased NLRP3 level from DCs, but is ineffective when P2X7 receptor was blocked (Fig. 3D). Our data verified combination of IR and CD39 inhibition on GSCs activated NLRP3 inflammasome of DCs in the way of P2X7 receptor dependent entry of eATP.

### Inhibition of STAT1 or CD39 enhanced activation of NF-κB p65-NLRP3-IL-1β pathway by eATP in DCs

We next detected p65-NLRP3-IL-1β inflammatory signaling pathway in DCs after IR. The results showed increased DCs engulfment, NLRP3 expression, and phosphorylation of NF-κB p65 after IR and CD39 inhibition (Fig. 3D–F). To confirm the role of NLRP3 in eATP-activated DCs, we used MCC950, NLRP3 inhibitor, to demonstrate the key role of p65-NLRP3-IL-1β pathway. NLRP3 inhibition decreased DCs engulfment (Fig. 3E) accompanying reduced NF-κB p65 and IL-1β expression (Fig. 3F). These data suggested that accumulated eATP by CD39 blockade activates NLRP3 inflammasome by P2X7 receptor in DCs (Fig. 3G).

**Fig. 4:**
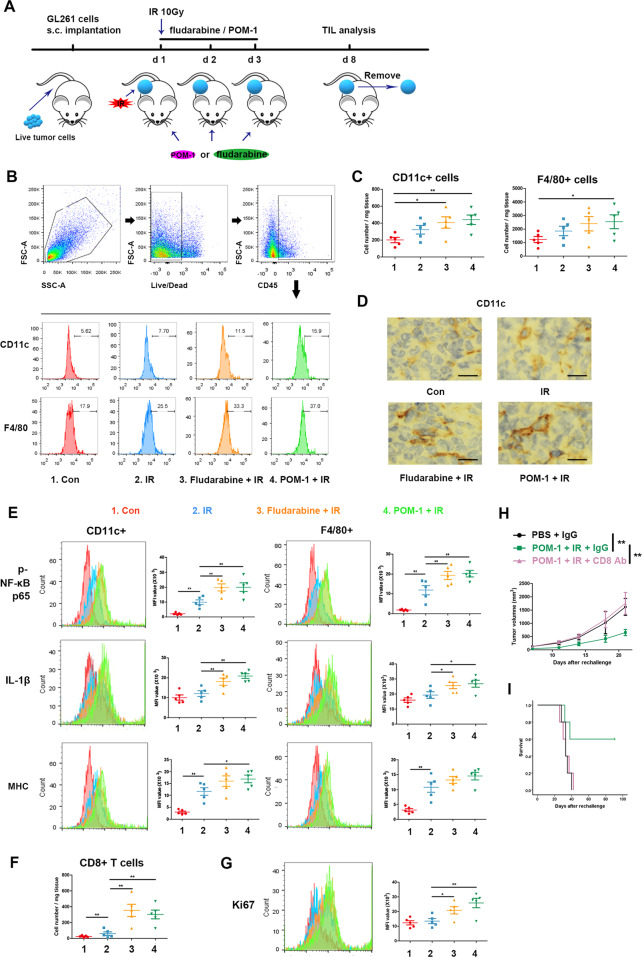
STAT1 or CD39 inhibition enhanced inflammasome activation of mononuclear phagocyte system in TME following IR. Subcutaneous tumors of C57BL/6 J mice were irradiated after mice were administrated with PBS, fludarabine, or POM-1, then were removed to prepare single-cell suspensions. Cells were stained for surface markers, then fixed and permeabilized for intracellular staining. Flow cytometry was used for markers measurement. **A** The schedule of treatment for mice is showed. **B** Representative plots showing the general gating strategy to delimit the positive CD45 cells from tumor tissue and positive CD11c and F4/80 cells in CD45+ T cells. **C** Positive CD11c and F4/80 cell number. **D** Positive CD11c cells were analyzed using IHC, and Images were captured with a light microscope (×400). Scale bars correspond to 20 μm. **E** The levels of phospho-NF-κB p65, IL-1β, and MHC in CD11c + or F4/80+ cells. **F** Positive CD8 cell number. **G** The levels of Ki67 in CD8 + cells. **H**, **I** The mice were removed from the untreated or IR + POM-1-treated tumors. were injected with live GL261s cells on the other side, and administrated with or without CD8 antibody. **H** the rechallenged tumor size was measured. **I** The survival of mice was recorded. *P < 0.05, **P < 0.01.

To further determine the activation of mononuclear phagocyte system in animal models, we investigated inflammatory signaling pathway following IR, STAT1 blockade, and CD39 inhibition. Flow cytometry showed the increase of DCs and macrophages in irradiated tumor tissues with the administration of STAT1 or CD39 inhibitor comparing to IR alone (Fig. 4B, C), and IHC consistently verified the increased number of infiltrating DCs (Fig. 4D). Combination of IR and STAT1 blockade or CD39 inhibition significantly increased the levels of p65 and IL-1β in NLRP3 inflammasome pathway and MHC in antigen presentation comparing to IR alone in DCs and macrophages (Fig. 4E), suggesting eATP, as “eat me” signal, activated innate immune cells by inducing more NLRP3 inflammasome generation. Meanwhile, the number and proliferation of CD8+ T cells in tumor tissues also increased (Fig. 4F, G).

To confirm if the immune effect was performed by CD8+ T cells in IR-induced ICD enhanced by CD39 inhibition, we depleted CD8+ T cells in C57BL/6J mice with subcutaneous tumor on the other side. Retarded tumor growth and prolonged survival, tumor-free and long survival in part, were found in the rechallenged mice in POM-1 and IR group, and were recovered to control after CD8 antibody administration (Fig. 4H, I). This confirms that IR induced ICD enhanced by CD39 inhibition cleared tumors by CD8+ T cells.

### CD39 expression has a negative correlation with immune cell infiltration levels in glioma patients

**Fig. 5:**
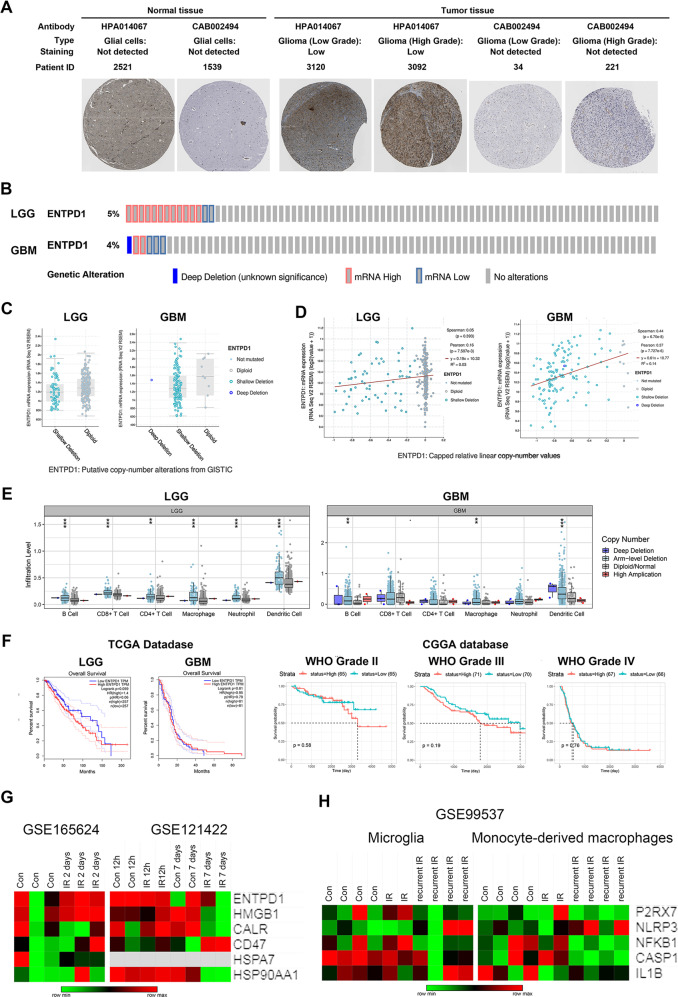
The correlation between CD39 alteration and immune cell infiltration in glioma. **A** The protein expression of CD39 using IHC in glioma patients from HPA database. **B** The changes in genome and mRNA of CD39 in TCGA dataset were showed. **C** The copy number alterations of CD39 gene were showed. **D** The relative analysis of CD39 copy number and mRNA expression value were showed. **E** Box plots showed the distributions of immune subset at each copy number status of CD39 gene. **F** Kaplan–Meier plots showed the differences between CD39^high^ and CD39^low^ patients’ survival from TCGA and CGGA databases. **G** Heatmap of mRNA-Seq analysis of ICD hallmarks expressions in different periods after radiation in GBM cell lines. **H** The indicated gene expression values in immune cell subtypes were depicted after radiation.

Next, we investigate the possible role of CD39 in GBM genesis and progress by bioinformatics analysis. Upregulated level of CD39 was showed by using IHC staining in the Human Protein Atlas (HPA) database (Fig. 5A). Furthermore, we analyzed the genomic, copy numbers, and mRNA expressions based on cBioPortal dataset from TCGA database, and the results in Fig. 5B showed 4–5% change of mRNA expression in glioma from TCGA dataset. Some samples in LGG and most samples in GBM harbored CD39 shallow deletion (Fig. 5C). The correlation of CD39 copy number value and mRNA expression is more relative in GBM samples (Fig. 5D). Then, we analyzed the potential relationship of CD39 expression and immune infiltration levels using TIMER web resource. The “SCNA” module showed immune cell infiltration levels in CD39 somatic copy number alterations, and the analysis demonstrated CD39 copy number was negatively relative with DCs infiltration (Fig. 5E). Moreover, the survival differences between CD39^high^ and CD39^low^ patients from TCGA and Chinese Glioma Genome Atlas (CGGA) databases were visualized using Kaplan–Meier plots (Fig. 5F). The differences were more obvious in LGG comparing with GBM probably due to more mRNA amplification and less gene shallow deletion showed in Fig. 5B–D.

Additionally, we assessed expressions of ICD hallmarks in patient-derived GBM lines using the downloaded GSE165624 dataset and in GBM cell line LN229 using downloaded GSE121422 dataset. Transcriptome analysis showed that ICD hallmarks were upregulated 48 h, however, downregulated 12 h and 7 days after radiation (Fig. 5G), suggesting that IR-induced ICD hallmarks expression may be a dynamic process. Meanwhile, we analyzed the expressions of extracellular ATP pathway in microglia and monocyte-derived macrophages by downloading GSE99537 dataset (Fig. 5H). No obvious changes in partial molecules were showed in heatmap since NLRP3 pathway activation needed NF-κB phosphorylation and Caspase-1 cleavage. The results demonstrated the activation of P2X7 receptor and NLRP3 pathway in mouse GBM model. The analyzed results from public database are consistent with our experiments. Upregulated CD39 in GSCs maybe resist the phagocytosis of immune cells on damaged cells after radiation, thereby performing immune escape.

### The stimulation of ICD-activated DCs effectively increases T cells activity

**Fig. 6:**
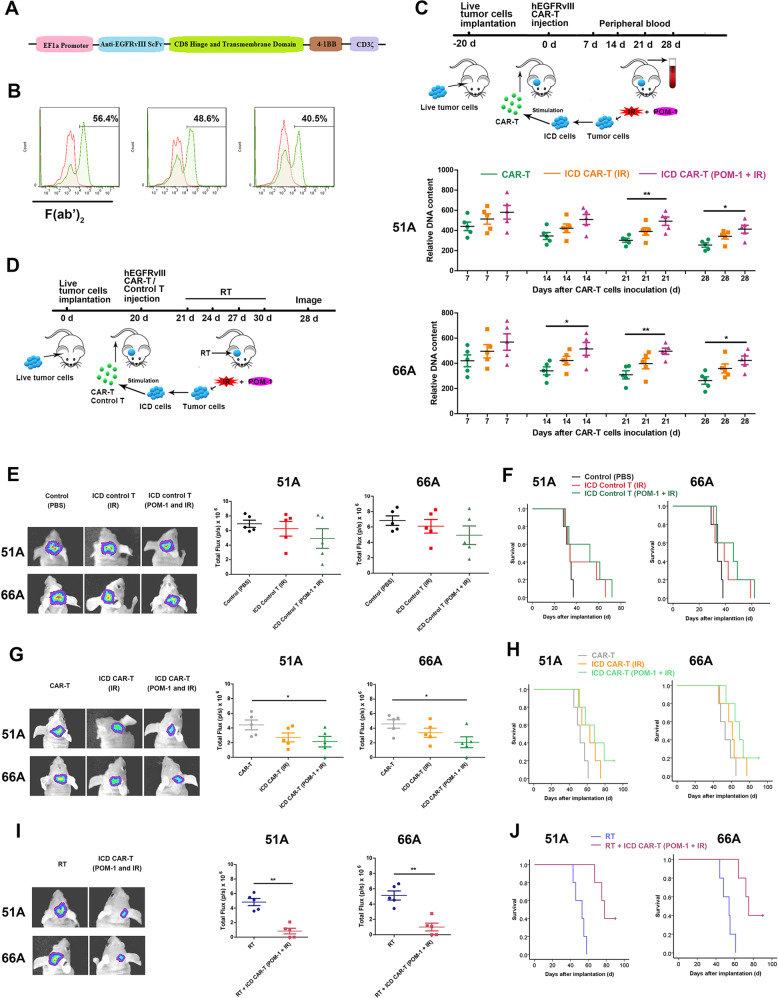
ICD-stimulating hEGFRvIII CAR-T cells improve efficiently antitumor efficacy. **A** The construction of EGFRvIII CARs was showed. **B** Expressions of CARs were confirmed by flow cytometry with F(ab’)2 antibody for human EGFRvIII. **C** ICD-activated hEGFRvIII CAR-T cells or CAR-T-cells were infused into the tail vein of nude mice with 51 A or 66 A xenografts. Peripheral blood was collected, and human genomic DNA in blood was detected using qPCR at an indicated time instead of CAR-T cells persistence in mice. *n* = 5. **D** Nude mice bearing tumor were injected with untransfected T cells or hEGFRvIII CAR-T cells with or without RT. The groups are following: 1. Control (PBS) group, the mice were injected with 100 μl PBS; 2. ICD Control T (IR) group, 51 A or 66 A cells were irradiated with 10 Gy and cultured for 4 days, then co-cultured with DCs for 24 hours. Untransfected T cells from healthy human PBMCs were added into culture system for 6 days. The activated T cells were administrated into mice via the tail vein on day 20 after 51 A or 66 A cells implantation; 3. ICD Control T (POM-1 and IR) group, GSCs were pretreated with POM-1 and irradiated, then treated as group 2; 4. CAR-T group, the mice were injected with hEGFRvIII CAR-Ts; 5. ICD CAR-T (IR) group, the hEGFRvIII CAR-Ts were treated as group 2; 6. ICD CAR-T (POM-1 and IR) group, the hEGFRvIII CAR-Ts were treated as group 3; 7. RT group, the mice were treated with RT 2 Gy once every 3 days for four times from day 21 after EGFRvIII transfected GSCs implantation. 8. RT plus ICD CAR-T (POM-1 and IR) group, the mice were treated with a combination of RT and hEGFRvIII CAR-Ts as group 6. Tumor size was measured by bioluminescence imaging signal (**E**) and the survival of mice was analyzed using Kaplan–Meier survival curves (**F**) after infusion of T cells from PBMCs. Tumor size (**G**) and survival (**H**) were evaluated after CAR-T infusion. The mice were treated by conventional RT after PBS or CAR-T injection, then tumor size (**I**) and survival (**J**) were evaluated after RT. *n* = 5. *P < 0.05, **P < 0.01.

We constructed an EGFRvIII CAR lentiviral vector transfected T-cell, and the expression of CARs was assessed by flow cytometry. The construction of CARs was shown in Fig. 6A, and the percentages of T cells positive for EGFRvIII CARs were 56.4%, 48.6%, and 40.5% in three infected CAR-T cells, respectively (Fig. 6B). Next, we tested the specificity of hEGFRvIII CAR-T cells lysing target cells. 51 A and 66 A cells with positive EGFRvIII expression (Supplementary Fig. 3A) were efficiently killed by hEGFRvIII CAR-T cells at an effector: target cells (E:T) ratio of 4:1. To determine the cytotoxic capacity of ICD-activated CAR-T cells against target cells, DCs were pulsed by irradiated GSCs with or without POM-1 pretreatment, then co-cultured by hEGFRvIII CAR-T cells. The CAR-T cells activated by DCs were incubated with target cells. The results showed that hEGFRvIII CAR-T cells activated by POM-1-pretreated and irradiated GSCs pulsing DCs (ICD CAR-T (POM-1 and IR)) compared with irradiated GSCs alone (ICD CAR-T (IR)) more efficiently lysed the target cells (Supplementary Fig. 3B).

Inflammatory cytokines secretion is another important factor in determining antitumor activity. ELISA assay demonstrated that more TNF-α, IFN-γ, IL-2 in detected two target cells and IL-10 in 51A cells were secreted by ICD CAR-T (POM-1 and IR) cells comparing to CAR-T cells or ICD CAR-T (IR) for 24 h co-culture with DCs activated by target cells at a ratio of 4:1 (Supplementary Fig. 3C). Furthermore, the concentration of perforin and granzyme B in the supernatants was also increased, which was consistent with the increase of cytotoxicity (Supplementary Fig. 3D). Taken together, these results indicated that activating CAR-T cells by ICD including CD39 inhibition in advance enhanced the cytotoxicity of EGFRvIII CAR-T cells.

### ICD-activated CAR-T cells showed effective and persistent antitumor activity against xenografts

For evaluating the persistence of CAR-T cells in vivo, the DNA content of the human genome in the venous blood of mice was detected using the human-specific GAPDH primer. The level of CAR-T cells in mice gradually decreased after infusion. The DNA content of CAR-T cells in mice treated with ICD-activated hEGFRvIII CAR-T cells including ICD CAR-T (IR) and ICD CAR-T (POM-1 and IR) was significantly higher than that in hEGFRvIII CAR-T cells treatment (Fig. 6C). Meanwhile antitumor activity of ICD-activated hEGFRvIII CAR-T cells was evaluated using orthotopic tumor model. Compared with CAR-T groups, the tumor size treated with ICD-activated hEGFRvIII CAR-T cells (POM-1 and IR) was progressively decreased (Fig. 6E, G), and the survival of mice bearing glioma was extended (Fig. 6F, H) in 51 A and 66 A model mice. There was one mouse still alive till 90-days, and no tumor was observed. The antitumor effect of the combination of RT and ICD-activated hEGFRvIII CAR-T cells was more significant compared to RT alone (Fig. 6I, J).

### Irradiated cells releasing ICD hallmarks as a vaccine enhance function of CAR-T cells in allograft model

**Fig. 7:**
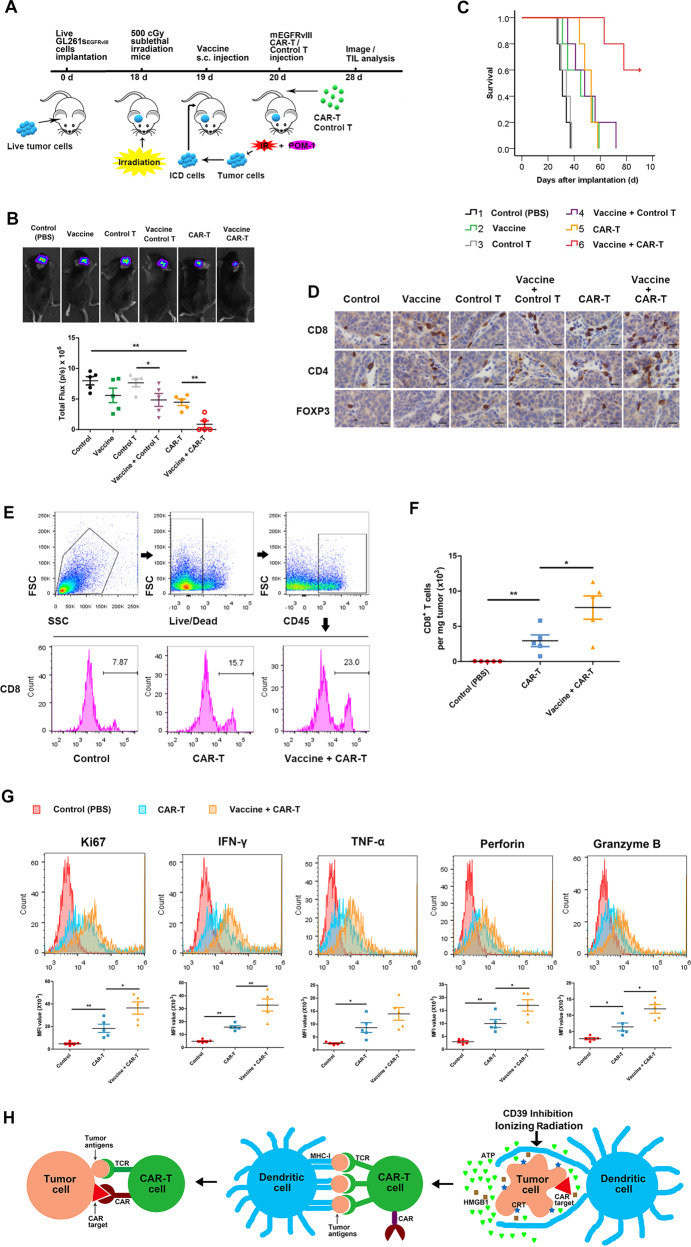
ICD hallmarks in vivo activated CAR-T cells showed persistent antitumor effect. C57BL/6 J mice bearing orthotopic GL261s_EGFRvIII_ glioma were treated with vaccine and mEGFRvIII CAR-Ts for antitumor therapy. The groups are following: 1. control group, the mice were injected with 100 μl PBS; 2. Vaccine group, GL261s cells were pretreated with 100 μM POM-1 for 3 hours, then irradiated with 10 Gy. The 10^7^-treated cells were inoculated s.c. into mice as a vaccine on day 19 after GL261s_EGFRvIII_ cells implantation; 3. Control T group, the mice were administrated with 10^7^ T cells from the spleen of healthy mice via tail vein on day 20 after GL261s_EGFRvIII_ cells implantation; 4. Vaccine plus Control T group, the mice were injected with vaccine and control T cells; 5. CAR-T group, the mice were administrated with 10^7^ mEGFRvIII CAR-Ts via tail vein on day 20; 6. Vaccine plus CAR-T group, the mice were injected with vaccine and CAR-Ts. **A** Schema of the mouse tumor model was used to test the effect of vaccine and CAR-Ts in vivo. Tumor size was measured (**B**) and the survival of mice (**C**) was analyzed. **D** Tumor-infiltrating T lymphocytes were measured using IHC stain for CD4, CD8, and FoxP3 (×400). Scale bars correspond to 20 μm. **E** Tumor was removed, and tumor-infiltrating CAR-T cells were examined by flow cytometry to evaluate the intratumoral expansion. **F** The number of positive CD8 T cells were calculated in tumor tissue. **G** Proliferative factor Ki67 produced by tumor-infiltrating CAR-T cells were analyzed by flow cytometry. **H** Tumor antigens are presented by DCs to CAR-T cells, then effective antitumor response is performed by multispecific CAR-T cells carrying CAR and tumor antigens targeting TCR. *n* = 5. *P < 0.05, **P < 0.01.

Activated CAR-T cells by ICD in vitro inhibited tumor growth and extended survival of mice bearing glioma, however, the therapeutic effect of ICD-activated CAR-T cells infusion in our experiment was limited. CAR-T cells activation in vivo will significantly promote CAR-T expansion [14]. Irradiated GL261s_EGFRvIII_ cells emitted ICD hallmarks, and POM-I pretreatment increased the level of ATP release post-irradiation (Supplementary Fig. 4). To verify ICD stimulation approach to boost CAR-T efficacy in mice in vivo, we prepared mouse T cells expressing murine EGFRvIII-specific (mEGFRvIII) scFv CAR (Supplementary Fig. 5A), which recognizes a short linear epitope of murine EGFRvIII. Immunization of mice expanded mEGFRvIII CAR-T cells in peripheral blood (Supplementary Fig. 5B–E). GL261s_EGFRvIII_ cells releasing ICD molecules as a vaccine were inoculated into lymphodepleted tumor-bearing mice for analysis of CAR-T therapeutic efficacy (Fig. 7A). Combination of CAR-T and vaccine significantly delayed tumor growth (Fig. 7B) and prolonged survival (Fig. 7C) of mice bearing allografts, and eliminated a part of tumors. IHC showed more CD4+ and CD8+ T cells infiltration in tumor tissue (Fig. 7D). In addition, inoculation of vaccine plus untransfected T cells also had a significant therapeutic impact. Flow cytometry showed that vaccine greatly increased CAR-T infiltration into tumors (Fig. 7E, F), and these TILs expressed higher levels of Ki67, IFN-γ, TNF-α, perforin, and granzyme B (Fig. 7G) than that of CAR-T cells therapy alone after 7 days post CAR-T infusion, suggesting increase of mEGFRvIII CAR-T cells polyfunctionality. Meanwhile, combination of vaccine and CAR-T enhanced antitumor efficacy of RT (Supplementary Fig. 6).

## Discussion

ICD hallmark stimulation is sufficient to release endogenous ATP to the extracellular environment with consequent activation of the P2X7 receptor, which allows the triggering of inflammasome response [29]. Recently, an accompanying study reported the antitumor immune effects of monoclonal antibody CD39, an extracellular ATP-converting enzyme, in solid tumor model [21]. Our data confirmed that IR-induced ICD hallmark release and CD39 inhibition-induced eATP increase activated NLRP3 inflammasome by P2X7 receptor in DCs, and NLRP3 activated inflammatory Caspase-1, which cleaved the pro-inflammatory cytokines pro-IL-1β and pro-IL-18 and induced the secretion of their active cytokines, consistent with the known NLRP3 activation mechanism by diverse stimuli [30]. These results indicated that IR upregulates CD39 expression via STAT1 activation, decreases the content of eATP and induces immunosuppressive TME in GSCs. ICD-generated cells as a vaccine provide a safe mean to activate the memory T cells and produce antigen-specific T-cell proliferation in vivo in normal homeostatic conditions [17]. Personalized neoantigen vaccine induces memory T-cell persistently [31], which has been confirmed by clinical trial from melanoma patients [32].

The vaccine can provide a potent strategy to elicit in vivo activation and expansion of CAR-T cells. In our research, culture of EGFRvIII CAR-T cells activated by ICD-generated cells with live GSCs resulted in secreting more cytokines and killing more tumor cells comparing with CAR-T cells alone. DCs pulsed with ICD hallmarks presented antigens to CAR-T cells, then activated CAR-T cells responded to antigens stimulation via the TCR, which made CAR-T cells expansion in vivo. Other researches also demonstrated CAR-T cells expansion when activated in vivo. Ma L and colleagues designed amphiphile CAR-T ligands to traffic to lymph nodes and decorated the surfaces of antigen-presenting cells, thereby CAR-T cells were activated in the native lymph node microenvironment [14]. Eyquem J et al. directed a CAR to the T-cell receptor α constant (TRAC) locus to induce uniform CAR expression in human peripheral blood T cells and enhance T-cell potency [33]. These data in our study demonstrated that the proliferative potential and killing ability of CAR-T cells were significantly enhanced, when memory CAR-T cells met with tumor cells again, due to tumor antigens presentation by DCs to CAR-T cells, suggesting that vaccine design for DCs activation should be an effective strategy to kill tumor cells by T cells proliferation. In addition, we confirmed ICD vaccine in vivo inoculation in Fig. 7 is a more effective strategy to activate CAR-T comparing to in vitro in Fig. 6 by the evaluation of CAR-T expansion and antitumor effect.

Antigen loss is a frequent cause of resistance to CAR-T immunotherapy [34, 35]. Mice with EGFR+ tumors were cured by EGFR CAR-T cells, and also rejected EGFR− tumors rechallenge, suggesting induction of epitope spread within CAR-T cells, but the extent and incidence of this phenomenon were unclear [36]. CAR-T cells provoked reversible antigen loss through trogocytosis, and the target antigen was transferred to T cells [37]. Effective antigens recognition is still common approach for enhancing CAR-T function. The designed vaccine presents whole antigens of tumor cells to CAR-T cells, which makes CAR-T cells achieving multispecificity besides CAR, to activate natural antitumor immune responses. Combinative therapy of the vaccine and CAR-T maybe overcame the obstacle of loss or downregulation of target antigens on the surface of a part of tumor cells by these mechanisms. Our strategy for enhancement of CAR-T cells effect against tumor bases on the theory of antigen spreading.

Taken together, we confirmed that IR-activated STAT1-IRF1 pathway regulates CD39 expression through IRF1-mediated CD39 transcription in radioresistant GSCs to generate immunosuppressive TME. CD39 blockade increases eATP post-IR in TME, then activates NLRP3 inflammasome by P2X7 receptor in DCs. Tumor antigens are presented by specific DCs to CAR-T cells, then effective antitumor response is mainly performed by CAR-T cells because of multispecificity of CAR-T cells including CAR and tumor antigens targeting TCR (Fig. 7H). Our study offered an effective immunotherapy strategy to demonstrate that a combination of the ICD vaccine and CAR-T cells therapy has the potential for tumor killing via direct activation of CAR-T cells in vivo.

## Materials and methods

### Ethics

The patient contributing to sectioned GBM tissues provided written informed consent to participate in the study, which was approved by the Ethics Review Board of the First Affiliated Hospital of Soochow University (No. 2018117).

### Cell culture

Human GSCs 51A, 66A, SHG141A, SHG142A from primary culture of GBM samples and mouse GSCs GL261s from mouse glioma cell line GL261 were cultured in serum-free DMEM/F12 medium containing supplement of 2% B27, 20 ng/ml epidermal growth factor and 20 ng/ml basic fibroblast growth factor. GSCs were isolated and identified as described previously [38], and all cells were incubated at 37 °C in humidified air with 5% CO_2_. These cell lines passed the mycoplasma test.

### Ionizing radiation

The cells were exposed to X-ray (160 KV) with a total dose of 10 Gy at room temperature using a linear accelerator (RadSource, Suwanee, GA, USA) at a dose rate of 1.15 Gy/min. IR on s.c. allografts were performed with 10 Gy X-ray irradiation (6 MV, the dose rate was 100 cGy/min) by a PRIMUS accelerator (SIEMENS Medical Solutions, Erlangen, Germany) once at room temperature. Radiotherapy (RT) on intracranial xenografts or allografts of mice was subjected to total 8 Gy X-ray with 2 Gy every 3 days using instrument as s.c. grafts. Irradiation was locally confined to the tumors by shielding the rest of the body with lead.

### Phagocytosis assays

Human blood collected from three healthy donors was separated by Ficoll-Hypaque gradient centrifugation to isolate peripheral blood mononuclear cells (PBMCs). PBMCs were divided into two parts, one half was used for DCs culture, and the other half was used for CAR-T cells preparation in later experiments. Monocytes were isolated from PBMCs by magnetic selection usingHuman CD14 Positive Selection Kit. The isolated cells were cultured in RPMI 1640 with 10% FBS with 50 ng/ml GM-CSF and 25 ng/ml IL-4 for 5 days. Immature DCs (iDCs) were stained with CFSE, and GSCs were stained with Far Red to measure phagocytosis. 10^5^ target cells were co-cultured at a 1:1 ratio with immature DCs for 24 hours, then cells were collected and analyzed using flow cytometry. DCs phagocytosing target cells were double positive cells of CFSE and Far Red.

### Vector transduction

GL261s cells were transfected with recombinant lentivirus vector targeting mouse EGFRvIII cDNA sequences as simulating the mutant mode of human gene. Plasmid carrying STAT1, IRF1, or STAT1-gRNA (Sequences in Supplementary Table 1) were transfected into GSCs. Cells were transfected with scramble sequences as a control.

### Specific site deletion

For the transient reporter analyses, CD39 promoter containing 1000 bp was amplified by PCR, and obtained fragments were cloned into the pGL3 Vector. Specific deletion of the putative IRF1 binding site was performed by the Q5 Site-Directed Mutagenesis Kit according to producer’s direction. The primers were in Supplementary Table 1.

### Luciferase reporter assay

GSCs were transfected with pGL3-CD39 promotor wild type (pGL3-CD39 promotor WT) or pGL3-CD39 promotor with specific deletion of the putative IRF1 binding site (pGL3-CD39 promotor IRF1∆) plasmid. Relative luciferase units were measured in IRF1 plasmid transfected or irradiated cells using the Dual-Glo Luciferase Assay System according to the manufacturer’s instructions. Relative luciferase units from firefly luciferase signal were normalized using Renilla signal.

### CRISPR/Cas9 gene knockout

The gene product from GSCs was deleted using CRISPR/CAS9 technology. Cells were transfected with lentivirus vector encoding guide RNAs (gRNA), and two guide sequences were shown in Supplementary Table 1.

### CAR vector design

The EGFRvIII targeting domain was synthesized and cloned into a CAR-encoding lentivirus backbone, named EGFRvIII CAR, containing a CD8 hinge spacer, transmembrane domain 4-1BB, and CD3ζ endo-domains. The EGFRvIII CAR was cloned and packaged into the lentivirus and expressed under the control of an EF-1a promoter. CARs directed to EGFRvIII antibodies single-chain variable fragments (scFvs) derived from human 139 and murine 3C10 antibodies were constructed for the transfection of human and murine-isolated T cells [6].

### CAR-T-cell preparation

T lymphocytes from PBMCs were purified by EasySep™ Human T Cell Isolation Kit, and cultured in RPMI 1640 containing 10% FBS with 300 U/ml IL-2. T cells were infected by human EGFRvIII CAR lentivirus, then co-cultured with DCs pulsed by irradiated glioma cells 4 days later. The human EGFRvIII CAR-T cells (hEGFRvIII CAR-Ts) were maintained at the density of 10^6^ cells/ml, and used for antitumor immune response study 10 days after transfection.

Mouse T cells were enriched from splenocytes using EasySep™ Mouse T Cell Isolation Kit. For T cells activation, anti-CD3 monoclonal antibody was pre-coated overnight at 4 °C, and anti-CD28 monocolonal antibody was added into the medium. Mouse T cells were infected by murine EGFRvIII CAR lentivirus to produce murine EGFRvIII CAR-T cells (mEGFRvIII CAR-Ts).

### Animal experiments in vivo

All animal experimental protocols were approved by the Institutional Animal Care and Use Committee of Soochow University and complied with the code of ethics for animal experiments. Male C57BL/6 J and nude mice (18–20 g) were bred and maintained in the Specific Pathogen-Free Animal Care Facility. The mice were randomly divided into groups after the formation of intracranial or s.c. grafts.

To analyze the activation of immune system from combination of IR and STAT1 or CD39 blockade, the 10^6^ GL261s cells were injected into right flank of C57BL/6 J mice in 100 μl PBS to establish s.c. allografts. When the tumor size reached a diameter of 6 cm, the mice were i.p. administrated with 100 mg/kg fludarabine or 12.5 mg/kg POM-1 for 3 days once every day, and the tumor were irradiated with 10 Gy locally on the first day. Tumors from mice were surgically removed for analysis of tumor-infiltrating lymphocytes (TIL) on the eighth day after IR (Fig. 4A). The mice were rechallenged with 10^6^ GL261s cells in 100 μl on left flank on the ninth day, and tumor size and survival were recorded. For CD8 T-cell blockade, 20 μg of CD8 or IgG in 100 μl was i.p. administered three times per week for three weeks on the day of tumor cell rechallenge.

For evaluation of antitumor effect of multispecific CAR-T cells therapy, the 10^5^ 51 A cells or 66 A were implanted into the frontal lobe of the mouse cerebrum of BALB/c nude mice by stereotactic implantation to establish intracranial xenografts. DCs were firstly pulsed by irradiated GSCs with or without POM-1 pretreatment. 10^7^ control T or hEGFRvIII CAR-Ts with or without DCs stimulation in 100 μl PBS were administrated by tail vein on day 20 post-implantation. The mice were performed conventional radiation therapy (RT) from day 21. A bioluminescence imaging signal was used to quantify the size of grafts at day 28. Mice were monitored daily until severe neurological deficits appeared, and survival analysis was used to compare the differences of each group according to survival time. The indicated treatment schedules were given in Fig. 6D.

For effect evaluation of ICD antitumor vaccine, 10^5^ GL261s_EGFRvIII_ cells were implanted into the frontal lobe of the mouse the cerebrum of C57BL/6 mice to establish intracranial allograft models. 500 cGy sublethal irradiation was carried out for lymphodepletion 18 days after glioma cells injection. The mice bearing tumor were s.c. immunized with 10^7^ irradiated GL261s cells with POM-1 pretreatment in 100 μl PBS as a vaccine on day 19. 10^7^ control T or mEGFRvIII CAR-Ts in 100 μl PBS were i.v. infused via the tail vein into recipient mice next day after vaccine inoculation. Tumor size and survival were recorded, and TIL was analyzed on day 28 (Fig. 7A).

### Intracellular cytokine staining

Tumors from mice were surgically removed, and single-cell suspension was obtained. Tumor-infiltrating immunocytes were analyzed after mechanical tumor dissociation. Tumors were dissected into 2–3 mm^3^ pieces and dissociated using gentleMACS Dissociator (Miltenyi Biotec). Cells were stained after red cell lysis and passing through a 70 μm cell strainer.

Cytokine production from TILs was investigated by stimulation with 50 ng/ml phorbol 12-myristate 13-acetate and 1 μg/ml of the calcium ionophore A23187. The protein transport inhibitor Golgiplug^TM^ was added before incubation in 5% CO_2_ at 37 ^o^C for 5 hours. Cells were stained using CD11c, F4/80, CD45, CD8, and/or MHC-I antibodies for 30 minutes at 4 °C, then fixed, permeabilized, and stained for intracellular cytokines or proteins. Flow cytometry was performed for tumor-infiltrating immunocytes analysis.

### Statistical analysis

Statistical analyses were performed using GraphPad Prism 5 and SPSS 10.0. All samples in vitro were replicated three times. Pair comparisons use student’s *t* test, and multiple comparisons use one-way ANOVA or two-way ANOVA with Tukey’s test. Animal survival was analyzed using Log-rank test.

## Supplementary information

Supplementary material 1 (supplementary Figures, methods, and Tables)

Supplementary material 2 (Original western blots)

Reproducibility checklist

## Data Availability

All datasets presented in this study are included in the article.
