# Supplementary material for: Enhanced radiation-induced immunogenic cell death activates chimeric antigen receptor T cells by targeting CD39 against glioblastoma
Source: Cell Death Dis. 2022 Oct 16;13(10):875. doi: 10.1038/s41419-022-05319-1 (PMC9573869; doi:10.1038/s41419-022-05319-1)
Supplement: Supplementary file 1 — Supplementary material 1 (supplementary Figures, methods, and Tables) [file 41419_2022_5319_MOESM1_ESM.docx]

**Enhanced radiation-induced immunogenic cell death activates chimeric antigen receptor T cells by targeting CD39 against glioblastoma**

Ting Sun^1^**^*^**, Yanyan Li^1^, Ying Yang ^2^, Bin Liu^1^, Yufei Cao^1^, Wei Yang ^2^**^*^**

1. Neurosurgery and Brain and Nerve Research Laboratory, The First Affiliated Hospital of Soochow University, Suzhou, Jiangsu, 215006, China

2. State Key Laboratory of Radiation Medicine and Protection, School of Radiation Medicine and Protection and Collaborative Innovation Center of Radiation Medicine of Jiangsu Higher Education Institutions, Soochow University, Suzhou, Jiangsu, China.

*Corresponding Authors: Ting Sun, E-mail: [sunting1979st@aliyun.com](mailto:sunting1979st@aliyun.com); Wei Yang, E-mail: detachedy@aliyun.com

**Supplementary Figures**


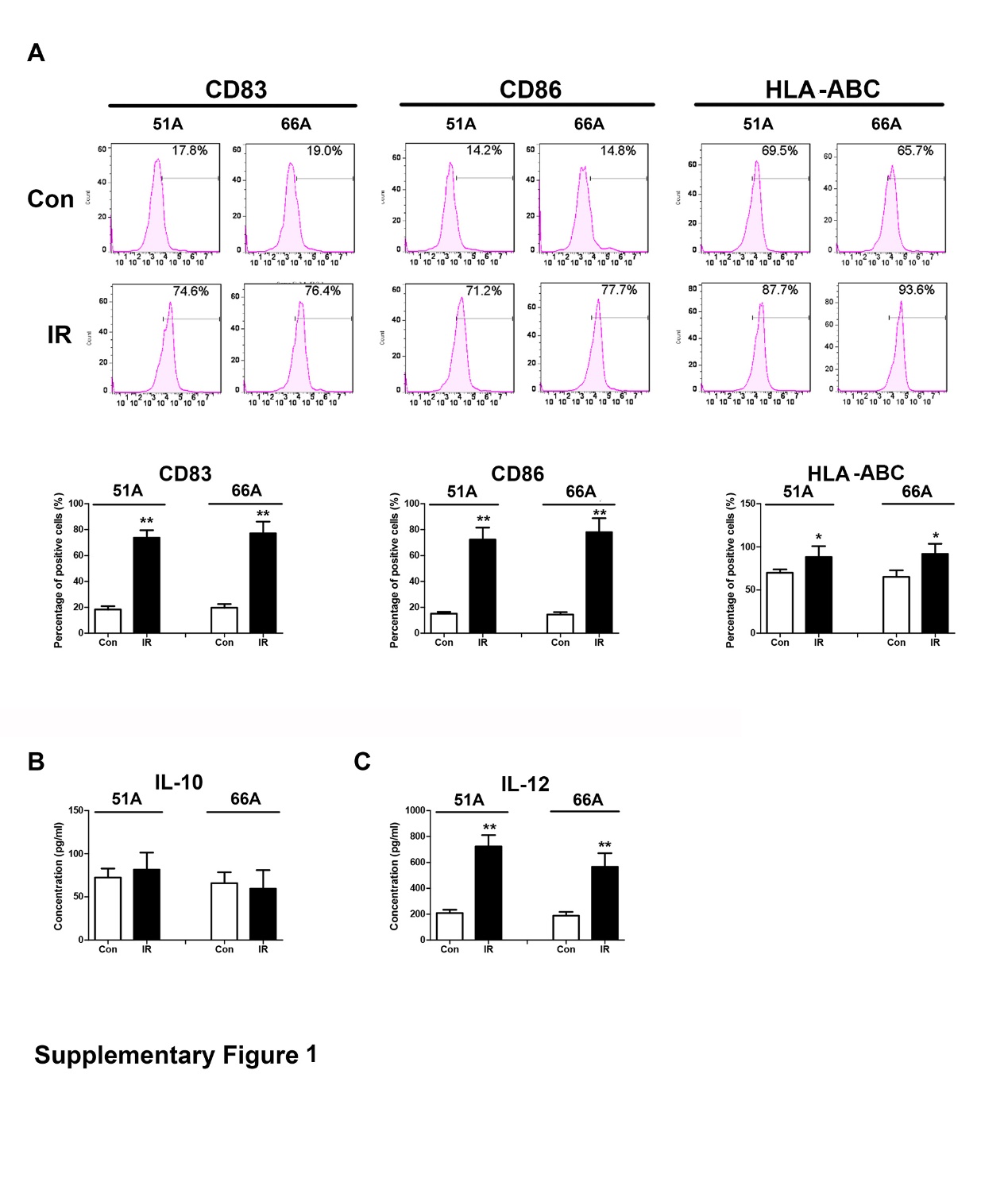


**Supplementary Figure 1. IR treatment induces mature and proinflammatory phenotype in DCs.** Untreated or irradiated GSCs cells were co-cultured with DCs for 24 hours at a 1:1 ratio. (A) The co-stimulatory markers CD83, CD86 and HLA in DCs were assessed by flow cytometry. The levels of IL-10 (B) and IL-12 (C) in cultured supernatants were measured by elisa. **P* < 0.05, ***P* < 0.01 vs control.


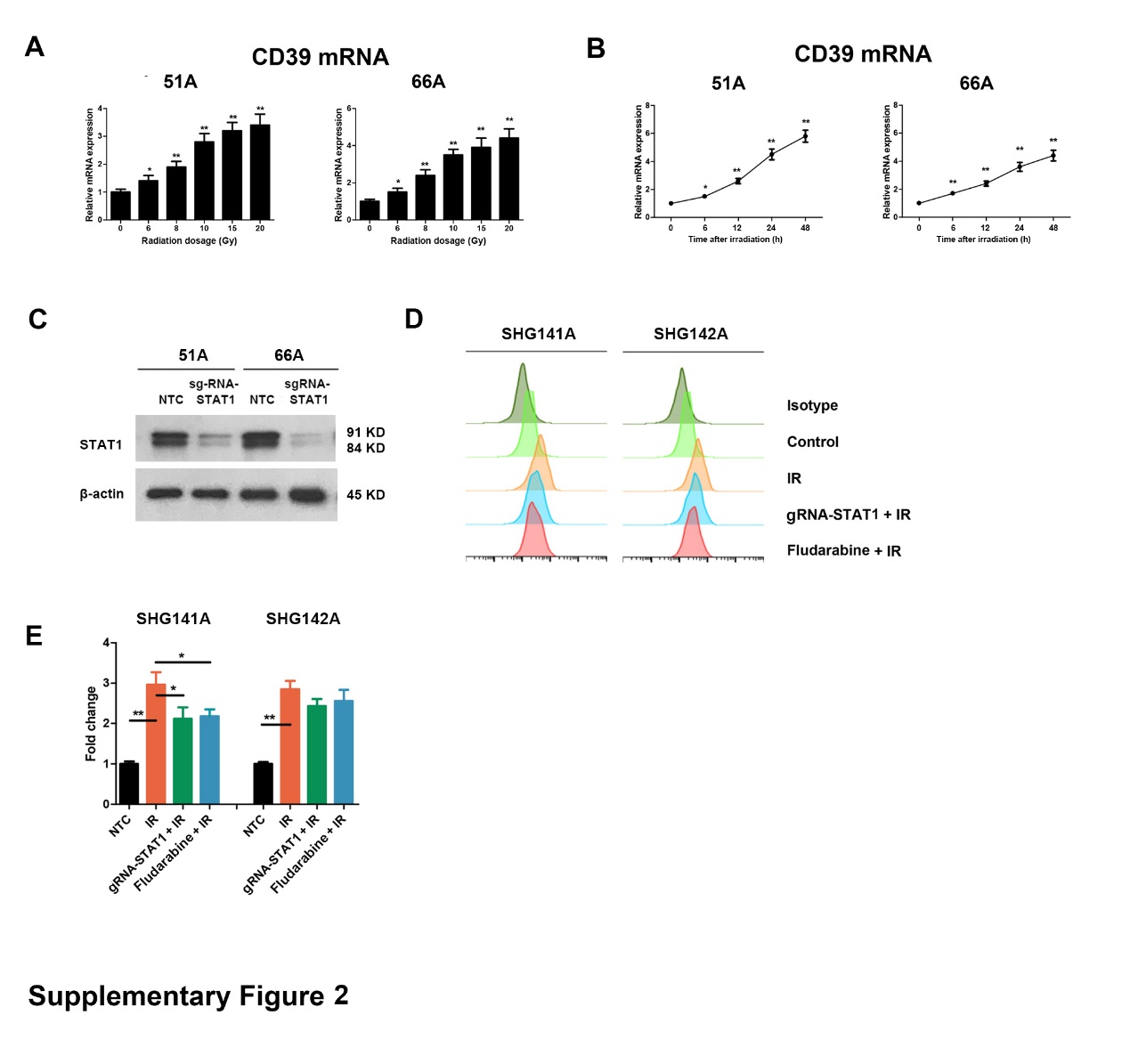


**Supplementary Figure 2. STAT1 regulates CD39 expression following IR.** (A) Cells were irradiated with indicated dosage, and cultured for 4 days. CD39 mRNA expression was detected using RT-qPCR, and GAPDH *w*as used as a reference gene. **P* < 0.05, ***P* < 0.01 vs 0 Gy. (B) Cells received 10 Gy IR and were cultured for different days. CD39 mRNA expression was detected. **P* < 0.05, ***P* < 0.01 vs day 0. (C) Cells were transfected with gRNA targeting STAT1, and STAT1 protein level from cell lysate were measured using western blot. Cells were transfected with gRNA-STAT1 or pretreated with 30 μM fludarabine for 6 h, then were irradiated with 10 Gy and cultured for 4 days. The level of CD39 protein on cell surface (D) using flow cytometry and mRNA expression (E) using RT-qPCR were detected. **P* < 0.05, ***P* < 0.01.


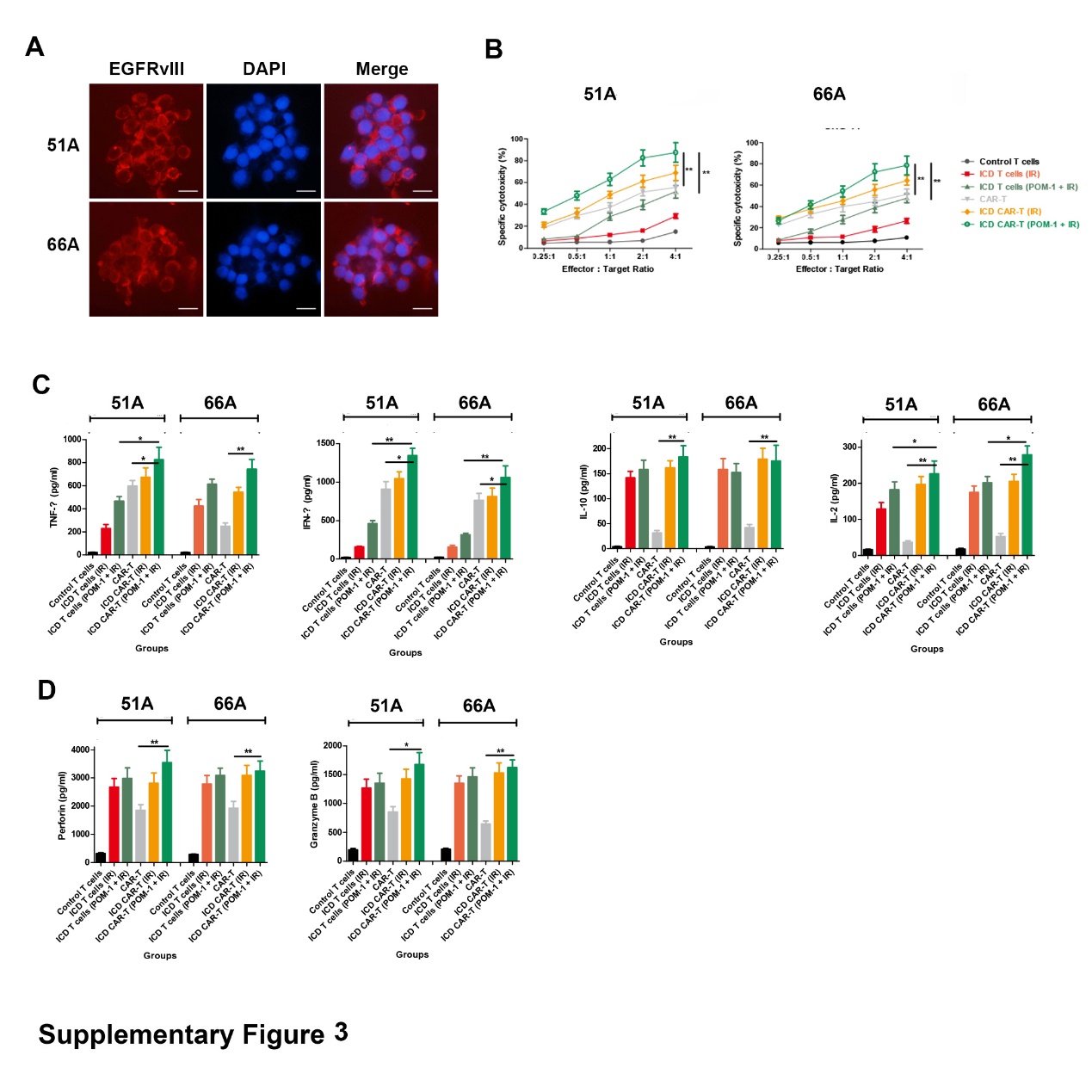


**Supplementary Figure 4. ICD activation efficiently induces phagocytosis of DCs and enhances the cytotoxicity of EGFRvIII CAR-T cells targeting EGFRvIII positive GSCs.** The hEGFRvIII CAR-Ts were co-cultured with DCs pulsed by untreated or irradiated GSCs with or without POM-1 pretreatment. The activated CAR-T cells were added into EGFRvIII positive cells for 24 hours. Homologous control T cells are used as a control. The groups are following: 1. Control T, isolated T cells are from PBMCs; 2. ICD T cells (IR), T cells were activated with DCs pulsed by irradiated GSCs; 3. ICD T cells (POM-1 + IR), T cells were activated with DCs pulsed by POM-1 pretreated and irradiated GSCs; 4. CAR-T, T cells were transfected with hEGFRvIII CAR lentivirus; 5. ICD CAR-T (IR), hEGFRvIII CAR-Ts were activated with DCs pulsed by irradiated GSCs; 6. ICD CAR-T (POM-1 and IR), hEGFRvIII CAR-Ts were activated with DCs pulsed by POM-1 pretreated and irradiated GSCs. (A) EGFRvIII expression was detected using immunofluoresence staining (×400 magnification). Scale bar =10 μm. (B) Control T or CAR-T cells were added into target cells culture system at indicated ratio of effector : target (E:T), and specific cytotoxicity was measured. (C) The levels of released cytokine in supernatants were measured using ELISA when T or CAR-T cells were co-cultured with target cells at an E:T ratio of 4:1. (D) The levels of perforin and granzyme B in the supernatantS of the co-culture system were measured by EKISA. Results represent average values derived from three different donor T cells. **P* < 0.05, ***P* < 0.01 vs control.


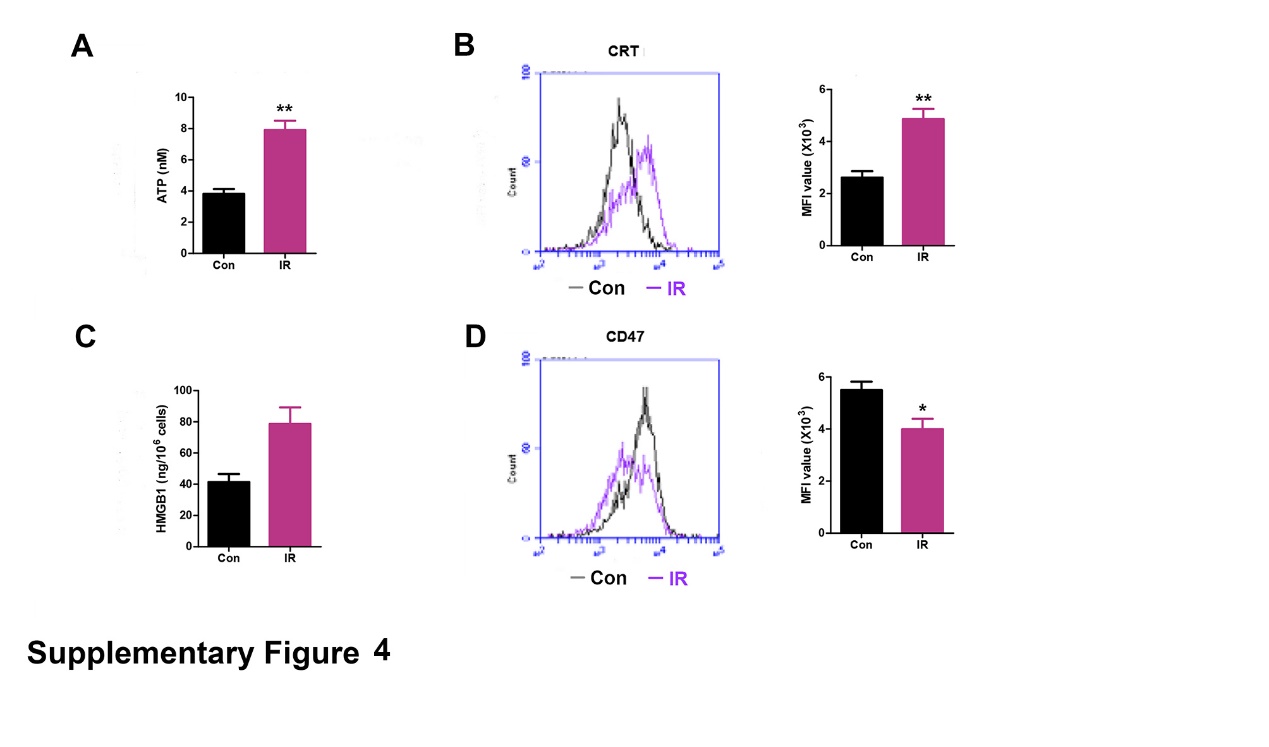


**Supplementary Figure 4. ICD hallmarks were exposed in murine GL261s cells after high dose IR.** GL261s cells were irradiated with 10 Gy, then ATP (A) in supernatant, the expression of CRT on cell surface (B), HMGB1 in supernatant (C) and CD47 expression on cell surface (D) were detected 24 hours later.


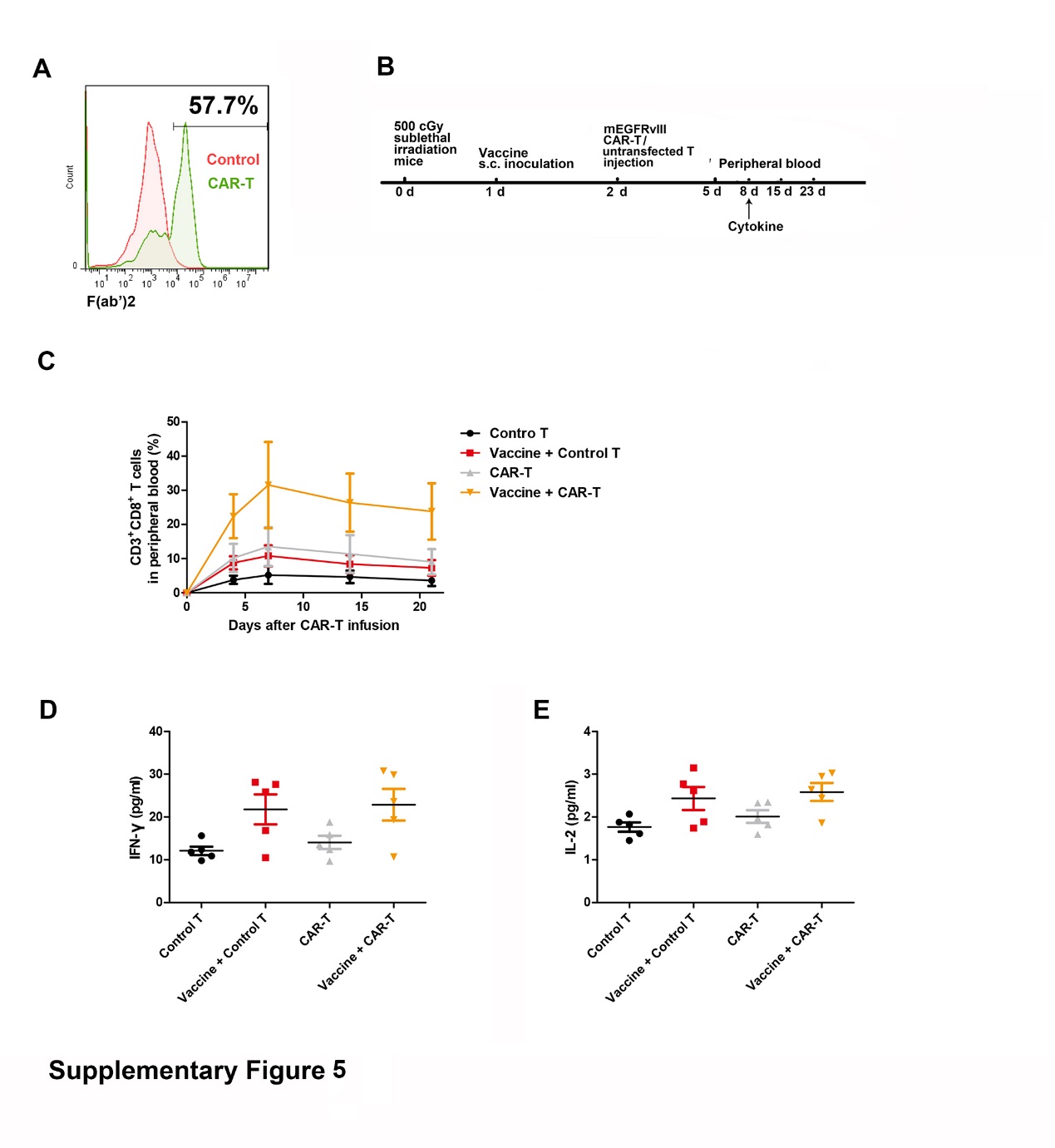


**Supplementary Figure 5. ICD hallmarks *in vivo* activated CAR-Ts for promoting expansion.** (A) Mouse EGFRvIII CAR-Ts were constructed, and expression of CARs was confirmed by flow cytometry. (B) Schema of the mouse was used to test CAR-Ts proliferation and function *in vivo*. C57BL/6J mice were irradiated with 500 cGy for lymphodepletion. GL261s cells with POM-1 pretreatment and IR were inoculated s.c. into mice as a vaccine. The mice were injected with 10^7^ untransfeted T or mEGFRvIII CAR-Ts next day. (C) Peripheral blood was collected at indicated time point, and CD3+ and CD8+ T cells were detected. (D) Cytokines in mice plasma were measured using an elisa assay, n=5. **P* < 0.05, ***P* < 0.01.


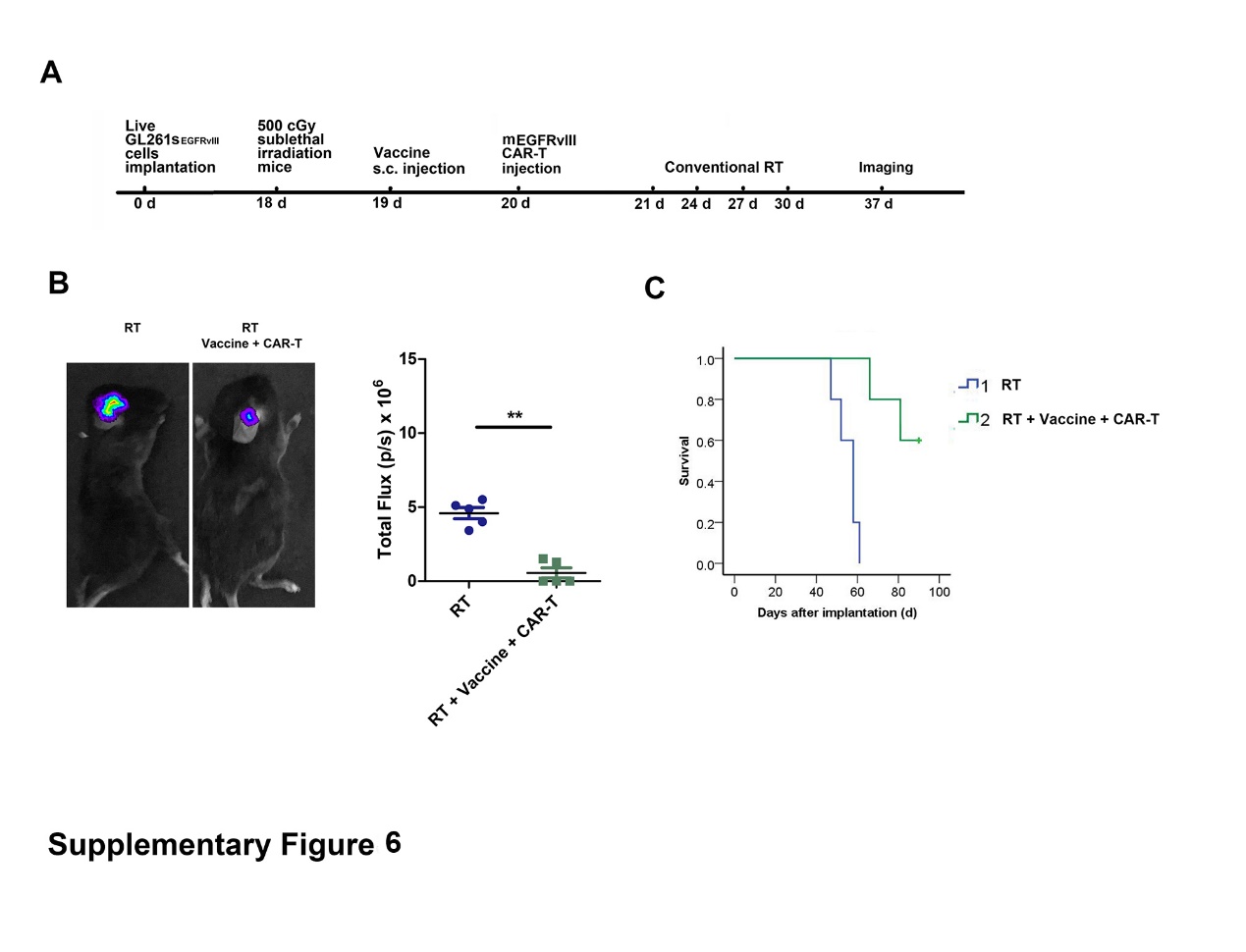


**Supplementary Figure 6. Combinative administration of vaccine and CAR-Ts enhanced antitumor effect of RT. (A)** C57BL/6J mice implanted with GL261s_EGFRvIII_ cells were administrated with PBS or vaccine plus mEGFRvIII CAR-Ts, then treated by conventional RT. Tumor size (B) and survival (C) were showed. n=5. **P* < 0.05, ***P* < 0.01.

**Supplementary Materials and Methods**

**Detection of DCs maturation**

Immature DCs were pulsed by irradiated or nonirradiated glioma cells for 24 h, then phenotypical assessment of DCs was determined by ﬂow cytometry using ﬂuorescein labelled monoclonal antibodies to HLA, CD83 and CD86.

**Cytotoxicity and cytokine secretion assay**

The antitumor activity of EGFRvIII CAR-T cells was evaluated using cytotoxic assay. Target cells 51A and 66A were seeded into 96-wells at a density of 4 × 10^4^ cells/well, and labeled with CFSE. After 24 h of co-culture with effector cells (DCs-activated untransfected T or hEGFRvIII CAR-T cells), the cells were collected, and dead cells were stained with 7-AAD and quantified by flow cytometry. Double positive cells of CFSE and 7-AAD were dead target cells. The supernatant of co-culture system was measured when effector cells were co-cultured with target cells at an effector: target (E:T) ratio of 4:1. The concentrations of perforin and granzyme B and the levels of cytokine secretion including IL-2, IL-10, TNF-α, and IFN-γ in the supernatant of co-culture system were measured using ELISA kits according to the manufacturer’s instructions.

**Immunofluoresence staining**

Cells were blocked with 5% bovine serum albumin after fixation with 4% paraformaldehyde. Primary anti-EGFRcIII antibody was used on cells overnight at 4°C, then secondary antibody was anti-rabbit Alexa Fluor 555. Cells were counterstained with DAPI and analyzed by fluorescence microscopy.

**DNA isolation, reverse transcription and quantitative polymerase chain reaction (RT-qPCR)**

Venous blood of nude mice was collected weekly for CAR-T cells assessment. Genomic DNA from 50 μl venous blood was isolated using a blood DNA kit. Total RNA was isolated from cells using Trizol reagent, and reverse transcribed into cDNAs using RevertAid First Strand cDNA Synthesis Kit. The DNA and cDNA content were analyzed using the SYBR Green PCR Master Mix on StepOne real-time PCR instrument (Applied Biosystems, USA). Human specific GAPDH as human DNA was detected in the extracted whole DNA. The relative mRNA levels were calculated by 2^−ΔΔCT^ method.

**Chromatin immunoprecipitation (ChIP)**

ChIP assay was performed using  [SimpleChIP](https://www.cellsignal.cn/product/productDetail.jsp?productId=9002)^[®](https://www.cellsignal.cn/product/productDetail.jsp?productId=9002)^[Enzymatic Chromatin IP Kit](https://www.cellsignal.cn/product/productDetail.jsp?productId=9002) according to the manufacturer’s instructions. In brief, cells were treated with formaldehyde and glycine, then 150-900 bp DNA fragments were achieved after lysing and ultrasonic treatment. The immunocomplexes were precipitated by using antibodies against IRF1 and subsequently pulled-down by Protein G Agarose Beads. Immunoprecipitated DNA was eluted and determined using qPCR reactions.

**Flow cytometry**

The cells were rinsed twice with phosphate saline buffer (PBS), fixed for 15 min with 0.2% paraformaldehyde for 15 min. The cells were permeabilized with saponin for intracellular staining. Primary and second antibodies were reacted in the dark for 30 min at room temperature. The expression levels of labeled proteins were analyzed by a flow cytometic Beckton Dickinson FACScan (BD Biosciences), and data were analyzed using FlowJo Software version 7.2.

**ATP assay**

ATP release were measured 24 h after 5 × 10^5^ cells were irradiated with or without fludarabine or POM-1 pretreatment. Supernatants were collected, then eATP was measured using an ATP Assay Kit based on luminescent signal.

**Enzyme-linked immunosorbent assay (ELISA)**

100 μl peripheral blood was collected from C57BL/6J mice injected with untransfected T or mouse CAR-T cells with or without vaccine 7 days post-injection. The concentrations of HMGB1, perforin, granzyme B, IL-2, IL-10, IL-12, TNF-α, and IFN-γ in plasma or supernatants from cultured cells were measured using elisa kits following the manufacturer’s instructions.

**Immunohistochemical (IHC) staining**

Tumor tissues were ﬁxed in parafﬁn, imbedded and cut for 4 mm sections. The sections were stained using anti-mouse CD11c, CD4, CD8 and FoxP3 primary antibodies at 4 °C overnight and biotin-labeled secondary antibody for 30 min at 37 °C. The sections were incubated with streptavidin–horseradish peroxidase complex and final signal was developed using the 3,3′-diaminobenzidine substrate for microscope observation.

**Supplementary Table 1. Nucleic Acid Sequences Table**

| Names | Sequences |
| --- | --- |
| STAT1 gRNA | 5’-AAAGCTGGTGAACCTGCTCCAGG-3’  5’-TCTTGCTACAGCATAACATAAGG-3’ |
| CD39 Primers | forward 5′-AGGTGCCTATGGCTGGATTAC-3′  reverse 5′- CCAAAGCTCCAAAGGTTTCCT-3′ |
| Primers for IRF1 binding site deletion | forward 5’-CCAGTCTAGAGTGCAATG-3’,  reverse 5’- TCTCAAAAAAACAAAGAAAAAAC -3’ |
| CD39 ChIP Primers | forward 5′-TCATGTTTCCACCACCACAG -3′,  reverse 5′-GCAGAAGAATCGCTTGAACC -3′ |

**Supplementary Table 2. Product Table**

| **Product** | **Company** | **Product Number** |
| --- | --- | --- |
| Calreticulin (D3E6) XP® Rabbit mAb (PE Conjugate) | Cell Signaling | 19780S |
| CD47 Monoclonal Antibody (B6H12), FITC | eBioscience™ | 11-0479-42 |
| TAP1 Polyclonal Antibody | Invitrogen™ | PA5-110458 |
| TAP2 Polyclonal Antibody | Invitrogen™ | PA5-102438 |
| PSMB9 Polyclonal Antibody | Invitrogen™ | PA5-19384 |
| PSMB8 Polyclonal Antibody | Invitrogen™ | PA5-22290 |
| HIF-1α (D1S7W) XP® Rabbit mAb | Cell signaling | 36169 |
| HLA-ABC Monoclonal Antibody (W6/32), APC | eBioscience™ | 17-9983-42 |
| CD83 Monoclonal Antibody (HB15e), PE | eBioscience™ | 12-0839-42 |
| CD86 (B7-2) Monoclonal Antibody (GL1), PE | eBioscience™ | 12-0862-82 |
| Phospho-NF-κB p65 (Ser536) (93H1) Rabbit mAb (PE Conjugate) | Cell signaling | 5733 |
| IL-1 beta (Pro-form) Monoclonal Antibody (NJTEN3), PE-Cyanine7, | eBioscience™ | 25-7114-82 |
| CD11c Monoclonal Antibody (N418), eFluor 450 | eBioscience™ | 48-0114-82 |
| F4/80 Monoclonal Antibody (BM8), eFluor 660 | eBioscience™ | 50-4801-82 |
| Ki-67 Monoclonal Antibody (SolA15), Alexa Fluor 700 | eBioscience™ | 56-5698-82 |
| CD8a Monoclonal Antibody (53-6.7), FITC, | eBioscience™ | 11-0081-82 |
| CD45 Monoclonal Antibody (30-F11), PE-Cyanine5.5 | eBioscience™ | 35-0451-82 |
| MHC Class I (H-2Kd) Monoclonal Antibody (SF1-1.1.1), APC-eFluor 780 | eBioscience™ | 47-5957-82 |
| Perforin Monoclonal Antibody (eBioOMAK-D), PE | eBioscience™ | 12-9392-82 |
| Granzyme B Monoclonal Antibody (NGZB), PE-Cyanine7 | eBioscience™ | 25-8898-82 |
| IFN gamma Monoclonal Antibody (XMG1.2), PerCP-Cyanine5.5 | eBioscience™ | 45-7311-82 |
| TNF alpha Monoclonal Antibody (MP6-XT22), eFluor 450 | eBioscience™ | 48-7321-82 |
| CD3 Monoclonal Antibody (17A2), eFluor 660 | eBioscience™ | 50-0032-82 |
| IL-1 beta Monoclonal Antibody (CRM56) | eBioscience™ | 14-7018-81 |
| NLRP3 Monoclonal Antibody (768319) | Invitrogen | MA5-23919 |
| Phospho-STAT1 (Tyr701) Monoclonal Antibody (ST1P-11A5) | Invitrogen | 33-3400 |
| Stat1 (D1K9Y) Rabbit mAb | Cell signaling | 14994 |
| IRF-1 (D5E4) XP® Rabbit mAb | Cell signaling | 8478 |
| β-Actin (13E5) Rabbit mAb | Cell signaling | 4970 |
| InVivoMab anti-mouse CD8α | BioXCell | BE0117 |
| InVivoMAb IgG2b isotype control | BioXCell | BE0090 |
| LIVE/DEAD™ Fixable Yellow Dead Cell Stain Kit | Invitrogen™ | L34967 |
| HMGB1 ELISA Kit | Arigo biolaboratories | ARG81351 |
| Human IL-2 Elisa Kit | Biolegend | 431807 |
| Human IL-10 Elisa Kit | Biolegend | 430607 |
| Human IFN-γ Elisa Kit | Biolegend | 430107 |
| Human TNF-α Elisa Kit | Biogems | [BGK01375](https://www.peprotech.com/zh/human-tnf%CE%B1-precoated-elisa-kit) |
| Mouse IL-2 Elisa Kit | Biolegend | 431007 |
| Mouse IL-10 Elisa Kit | Biolegend | 431417 |
| Mouse IFN-γ Elisa Kit | Biolegend | 430807 |
| Mouse TNF-α Elisa Kit | Biogems | [BGK06804](https://www.peprotech.com/zh/murine-tnf%CE%B1-precoated-elisa-kit) |
| [Human Perforin ELISA development kit](http://www.bioec.cn/product/6_1440978/MabTech_3465-1A-6_Human%2520Perforin%2520ELISA%2520development%2520kit%2520%28ALP%29_for%25206%2520plates) | MabTech | 3465-1A-6 |
| [Human Granzyme B ELISA development kit](http://www.bioec.cn/product/6_1440641/MabTech_3485-1A-6_Human%2520Granzyme%2520B%2520ELISA%2520development%2520kit%2520%28ALP%29_for%25206%2520plates) | MabTech | 3485-1A-6 |
| EasySep™ Human CD14 Positive Selection Kit II | STEMCELL Technologies | 17858 |
| EasySep™ Human T Cell Isolation Kit | STEMCELL Technologies | 17951 |
| EasySep™ Mouse T Cell Isolation Kit | STEMCELL Technologies | 19851 |
| ImmunoCult™ Human CD3/CD28 T Cell Activator | STEMCELL Technologies | 10971 |
| Anti-Mouse CD3e Antibody, Clone 145-2C11 | STEMCELL Technologies | 60015 |
| CD28 Monoclonal Antibody (37.51) | eBioscience™ | 14-0281-82 |
| Recombinant Human GM-CSF | PeproTech | 300-03 |
| Recombinant Human IL-4 | PeproTech | 200-04 |
| Recombinant Human IL-2 | PeproTech | 200-02 |
| Mouse IL-2 Recombinant Protein | Gibco | PMC0024 |
| CellTrace™ Far Red Cell Proliferation Kit | Invitrogen™ | C34564 |
| CellTrace™ CFSE Cell Proliferation Kit | Invitrogen™ | C34554 |
| [SimpleChIP^®^ Enzymatic Chromatin IP Kit](https://www.cellsignal.cn/product/productDetail.jsp?productId=9002) | Cell signaling | 9002 |
| Q5® Site-Directed Mutagenesis Kit | New England BioLabs | E0554S |
| Dual-Glo Luciferase Assay System | Promega | E2920 |
| RevertAid First Strand cDNA Synthesis Kit | Thermo Scientific™ | K1622 |
| PowerUp™ SYBR™ Green Master Mix | Applied Biosystems™ | A25742 |
| TRIzol™ Reagent | Invitrogen™ | 15596018 |
| Qiaamp dna blood mini kit | Qiagen | 51104 |
| Luminescent ATP Detection Assay | Abcam | Ab113849 |
| EGF Receptor vIII (D6T2Q) XP Rabbit mAb | Cell signaling | 64952 |
| phorbol 12-myristate 13-acetate | Sigma-Aldrich | P1585 |
| calcium ionophore A23187 | Sigma-Aldrich | 100107 |
| Fixation/Permeabilization Solution Kit with BD GolgiPlug™ | BD Biosciences | 555028 |
| Sodium metatungstate | MedChemExpress | HY-103259 |
| ACK Lysing Buffer | Gibco^TM^ | A1049201 |
