# Supplementary material for: Enhanced radiation-induced immunogenic cell death activates chimeric antigen receptor T cells by targeting CD39 against glioblastoma
Source: Cell Death Dis. 2022 Oct 16;13(10):875. doi: 10.1038/s41419-022-05319-1 (PMC9573869; doi:10.1038/s41419-022-05319-1)
Supplement: Supplementary file 2 — Supplementary material 2 (Original western blots) [file 41419_2022_5319_MOESM2_ESM.docx]

**Original blots**

Western blot in Figure 2D


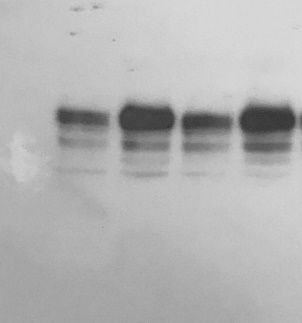

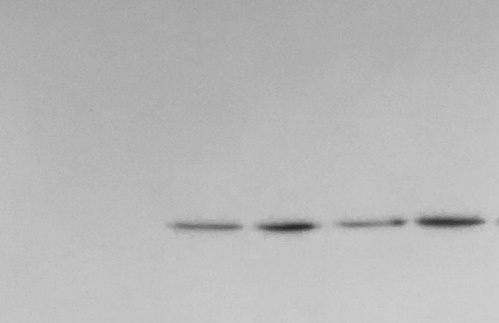




STAT1 IRF1 β-actin

Western blot in Supplementary Figure 2C




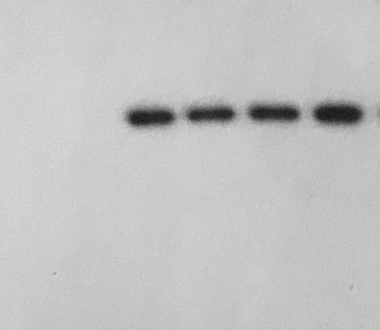


STAT1 β-actin
